# Vividness of visual imagery is associated with perceived clarity of recall but not with accuracy during exams

**DOI:** 10.3389/fpsyg.2026.1867712

**Published:** 2026-08-28

**Authors:** Zitian Xing, Andrea Nicholas

**Affiliations:** 1 Department of Psychology, Vanderbilt University, Nashville, TN, United States; 2 Department of Neurobiology and Behavior, University of California, Irvine, Irvine, CA, United States

**Keywords:** exam performance, learning preferences, neuroscience education, recall clarity, study approaches, VVIQ

## Abstract

**Introduction:**

Visual imagery may aid learning by facilitating vivid mental images, but it remains unclear whether greater imagery vividness improves students’ exam performance through enhanced visual recall clarity. This study examines the association between vividness of visual imagery and recall clarity during exams and evaluates their direct and indirect links to exam performance.

**Methods:**

Undergraduate students (*n* = 332) enrolled in a general education neuroscience course took two 25-item multiple-choice midterm exams. After the exams, they completed a structured survey assessing visual imagery ability via the Vividness of Visual Imagery Questionnaire (VVIQ) and visual learning preferences via the VARK questionnaire. For each randomly selected exam item, students reported which study approaches they recalled from and rated the clarity of their visual recall. Responses were linked to question-level answer correctness and overall exam scores.

**Results:**

Visual imagery ability and subjective recall clarity were positively correlated, but neither predicted answer correctness nor overall exam scores. A statistically significant association between VVIQ scores and overall GPA was observed, but its practical significance was negligible. Additionally, visual learning preference was statistically associated with recall clarity, although the small effect size and limited practical significance did not predict exam performance. Among reported study approaches, recall of lecture-based learning was associated with higher answer correctness than recall of online resources.

**Discussion:**

Taken together, visual imagery vividness, perceived recall clarity, and visual learning preference did not predict summative exam performance, whereas recall of lecture-based learning during the exam was associated with better performance. This suggests that visual imagery ability is not a major predictor of exam success and that keeping pace with in-class instruction and prioritizing classroom learning are more closely related to better outcomes.

## Introduction

In many large undergraduate science courses, closed-book, time-limited exams are the primary form of summative assessment (Rummer et al., 2019). When external materials are unavailable in these situations, students often rely on internally generated images (the mind’s eye) to reconstruct diagrams, spatial relationships, and procedural sequences needed to answer exam questions (Roach et al., 2020; Baade et al., 2025; Zhao et al., 2025). It is important to elucidate whether the clarity of visual recall helps students perform better on exams, both for basic memory research and for decisions about teaching and designing exams.

Visual imagery is the cognitive process of creating, manipulating, or recalling vivid mental pictures in the mind’s eye without direct external visual stimulation. It plays a central role in how people encode and retrieve visual information (Pearson, 2019). Cognitive and educational research has established substantial benefits for certain learning processes, especially retrieval practice, spaced repetition, and learner-generated representations such as drawing (Agarwal et al., 2021; Price et al., 2025; Dechamps and Skulmowski, 2025). Related work indicates that individual differences in imagery vividness, commonly measured by the Vividness of Visual Imagery Questionnaire (VVIQ), relate to performance on spatial tasks, object memory, and creative problem solving (Gu et al., 2025). Visual imagery vividness can aid learning; evidence shows that students who can create clearer visual images are more successful at recalling complex visual information during exams, particularly when they must mentally manipulate or reconstruct what they have learned (Bobek and Tversky, 2016; Gjorgieva et al., 2023). However, vividness does not reliably predict classroom achievement or overall academic success. Previous domain-specific studies report mixed task-dependent effects (Tabi et al., 2021; Zhou et al., 2024). Vivid visual imagery improves detailed episodic recall, comprehension during deep reading, and specific visuospatial problem-solving tasks; yet, it has poor or negligible correlations with general assessments, standardized examinations, and most routine learning activities (Lauren et al., 2025). Research on aphantasia finds that many learners who lack voluntary visual imagery rely on spatial processing or verbal/external compensatory strategies and often achieve comparable academic outcomes (Pounder et al., 2022; Reeder et al., 2024).

However, concurrent assessment of visual imagery ability, subjective clarity of visual recall during exams, and academic performance in large undergraduate courses have not been reported. At the same time, digital teaching tools and diverse learning approaches have quickly expanded to universities worldwide. The number of videos, slides, and online question banks that students can choose from has skyrocketed (Morgado et al., 2024; Fisher et al., 2024). Previous research found that practicing illustrating neuroscience concepts with the online scientific-illustration tool BioRender positively impacted student learning (Ha et al., 2024). These changes raise practical questions: in contexts of highly varied resources and approaches, how do individual differences (e.g., visual imagery vividness) and specific study behaviors jointly affect performance on real, timed, closed-book assessments? This omission leaves it uncertain whether visual imagery influences students’ exam performance by enhancing the clarity of visual recall on classroom assessments, and whether the types of study approaches students recall affect exam performance.

Learning style preferences, as defined by the VARK model (Visual, Auditory, Read/Write, Kinesthetic), may predict how students process and recall information (Elsaftawy et al., 2024). Visual learners, for instance, may have a natural ability to recall visual imagery, which could give them an advantage on exams without visual aids (Pearson, 2019). On the other hand, the belief that style preference improves performance is not well supported. Further, matching learning style to instructional method demonstrated no statistically significant improvements in students’ performance in immediate or delayed comprehension tests (Rogowsky et al., 2020; Clinton-Lisell and Litzinger, 2024; Hattie and O'Leary, 2025). This suggests that while learning style may shape how students process information, its effect on academic performance is uncertain.

The present study addresses these gaps by combining VVIQ scores, recall clarity during exams, visual learning preference (VARK), and exam performance in a large non-major undergraduate neuroscience course. Students were asked to complete the VVIQ to assess visual imagery ability and the VARK to assess visual learning preference. For each randomly selected exam question, students reported which study approaches they recalled from and rated the clarity of visual recall; exam items were scored as binary to allow source-level prediction of correctness. This design tests whether visual imagery ability correlates with recall clarity during exams and whether these measures directly or indirectly predict exam performance. By examining how vividness of visual imagery, recall clarity, and visual learning preference relate to outcomes on authentic classroom assessments, we aim to clarify cognitive mechanisms underlying successful test-taking in science education.

## Materials and methods

### Participants

A total of 332 undergraduate students participated in this study. All students were enrolled in a general education neuroscience course called Drugs and the Brain, which was designed for non-majors at the University of California, Irvine. This course fulfills a science and technology general education requirement for non-biology students. All participants provided informed consent and completed the study for extra credit. The population included 89 freshmen, 68 sophomores, 104 juniors, and 71 seniors. The enrolled majors of the student population varied widely across both traditional STEM and non-STEM disciplines but did not include any biology majors. Student primary majors consisted of: psychology (141), criminology, law and society (32), cognitive sciences (15), political science (11), engineering (10), business administration (9), sociology (8), pharmaceutical sciences (7), education sciences (7), languages (7), business economics (6), computer science (5), public health (5), anthropology (5), undeclared (24), and fewer than 5 students for each of the following: art, art history, chemistry, comparative literature, dance, drama, economics, environmental science and policy, film and media, history, international studies, physics, social ecology, urban studies.

### Procedure

All students first took two midterm exams, each including 25 multiple-choice questions designed to evaluate their conceptual understanding and application of the course materials. Then, a structured learning survey was administered to collect data and link it to midterm exam performance. The survey consisted of the following four sections:

#### Background information

Students were asked to provide general background information, including their college year, major, cumulative GPA, and weekly study time spent on the subject after class. Study time was measured with a 4-point scale: 1 representing less than 1 h, 2 representing 1–3 h, 3 representing 4–6 h, and 4 representing more than 7 h.

#### Visual imagery ability

Visual imagery, or the “mind’s eye,” is introduced with the following definition: “To define what a mind’s eye is, if you see something in your mind’s eye, you can imagine an item and clearly see it in your mind.” Students’ visual imagery ability was assessed with a 16-item VVIQ. The self-assessment tool consists of four groups of four questions, totaling 16 scenarios. Participants were asked to visualize a particular scenario, such as the face of a relative or friend you see often, a rising sun, the front of a familiar shop, or a beautiful landscape, and rate how vividly they can be visualized in mind based on a set of prompts. Each item was scored on a Likert scale ranging from 1 to 5. 1 = no image at all; you only know that you are thinking of the object. 2 = dim and vague; flat. 3 = moderately clear and lively. 4 = clear and lively. 5 = perfectly clear and lively, as in real seeing. The total VVIQ score ranges from 16 to 80 (Larner et al., 2024). The cutoffs of VVIQ score are defined as follows: 16 = aphantasia; 17–32 = hypophantasia; 33–74 = typical imagery ability; 75–80 = hyperphantasia (Wright et al., 2024).

#### Recall sources and visual recall clarity

Students were asked to reflect on how they recalled visual memories related to 16 multiple-choice questions randomly selected from the 50-item midterm exam. The same 16 questions were shown to all students. This subset was selected to keep the survey around 15–20 min, minimize participant burden and fatigue, and reduce missing responses or satisficing that might occur if all 50 items were rated. Random selection also provided an unbiased representation of the full exam while limiting content-specific bias. Across 332 students, this design yielded over 5,000 item-level observations, providing sufficient information for subsequent analyses. For each question, students reported which study approaches they were remembering when answering the exam item (e.g., discussing with other students, homework answers, a moment in class when they learned the content, content in their notes, reading the content online, or content on a class PowerPoint). They then rated how clearly they could see the corresponding visual mental image of this recall source in their mind during recall, using the same 5-point scale as the VVIQ. Recall clarity was defined as the subjective vividness and clarity of the mental image experienced during retrieval to answer an exam question. This measure was intended to capture state-level visual imagery at the time of recall and is conceptually distinct from trait-level visual imagery ability assessed with the VVIQ. Thus, recall source specifies what content was retrieved, whereas recall clarity indexes the quality of the associated visual mental imagery during retrieval. The average recall clarity of the 16 questions for each student was calculated. In our survey, the 16 sampled items were presented in a fixed common order (not randomized per student). To minimize memory decay and reconstruction bias, the survey was administered immediately after exam completion and was limited to 15–20 min. The original exam item stems were displayed to facilitate recall.

#### Visual learning preference

Learning style preference was assessed using the VARK questionnaire, which comprised 16 multiple-choice questions with 4 options for each (Elsaftawy et al., 2024). Each option relates to the preference for a precise sensory modality: visual, auditory, read/write, and kinesthetic. Students were allowed to choose more than one option for each question. The total number of visual responses on each completed VARK questionnaire was counted for each participant and used to represent their degree of visual learning-style preference.

### Measures and reliability

Internal consistency was assessed using Cronbach’s alpha, with 95% CIs estimated from 1,000 bootstrap resamples. Consistent with recommendations for evaluating educational and psychological measures, reliability evidence was examined for all multi-item scales used in the study (American Educational Research Association, American Psychological Association, and National Council on Measurement in Education, 2014). The VVIQ demonstrated excellent internal consistency [*α* = 0.9477, 95% CI (0.9365, 0.9589)]. The recall clarity scale, calculated across the 16 randomly selected exam items, also showed strong internal consistency [*α* = 0.9033, 95% CI (0.8841, 0.9224)]. For the VARK visual learning score (sum of visual options across 16 items), reliability for dichotomous items was acceptable [KR-20 = 0.760, 95% CI (0.720, 0.798)]. Item-total correlations were positive and within acceptable ranges for both measures.

### Statistical analysis

All statistical analyses were performed using Stata 18.0. Statistical significance was set at *p* < 0.05. We explored how individual differences in the vividness of students’ visual imagery (VVIQ scores), visual recall clarity, and study behaviors related to exam performance. Simple linear regressions were carried out, and scatterplots were created to show their relationship. Students were grouped into four groups based on their VVIQ scores. Answer correctness and total exam scores across visual imagery ability levels were compared using one-way ANOVA. A one-way ANOVA followed by *post hoc* pairwise comparisons and a chi-square test of independence were conducted to examine whether the recall sources were associated with exam performance. Prior to conducting linear regression analyses, model assumptions were evaluated using graphical and statistical diagnostics following standard recommendations for applied regression analyses (Hickey et al., 2019; Shatz, 2024). Residuals-versus-fitted plots assessed linearity and homoskedasticity (Supplementary Figure S1). Residual normality was evaluated with Q-Q plots and Shapiro–Wilk tests (Supplementary Figure S2 and Supplementary Table S2). Although some Shapiro–Wilk tests were significant, the corresponding Q-Q plots showed only minor deviations; given the large sample size (*n* = 332), these were not considered consequential for inference. Breusch-Pagan and White tests were used to assess homoskedasticity (Supplementary Table S2). For the model predicting cumulative GPA from VVIQ scores, the Breusch-Pagan test indicated heteroskedasticity; we therefore re-estimated this model using heteroskedasticity-robust standard errors. Overall, the diagnostics supported the use of linear regression for all models. To evaluate potential order effects, item position (1–16) was included as a predictor in supplementary analyses of recall clarity and answer correctness. Recall clarity was analyzed using a linear mixed-effects model, and answer correctness was analyzed using a mixed-effects logistic regression, with random intercepts for students and items. Descriptive statistics for the primary outcome variables are provided in Supplementary Table S1.

### Ethics statement

This study was reviewed and approved by the University of California, Irvine Institutional Review Board (Study ID: 20162910). It was conducted in accordance with the local legislation and institutional requirements. The study consisted of an anonymous survey of university students conducted as part of routine classroom activities and posed minimal risk to participants. Participation was voluntary, informed consent was obtained from all participants prior to participation, and no personally identifiable information was collected.

## Results

### Visual imagery ability and recall clarity are positively correlated, but neither predicts exam performance

**Figure 1:**
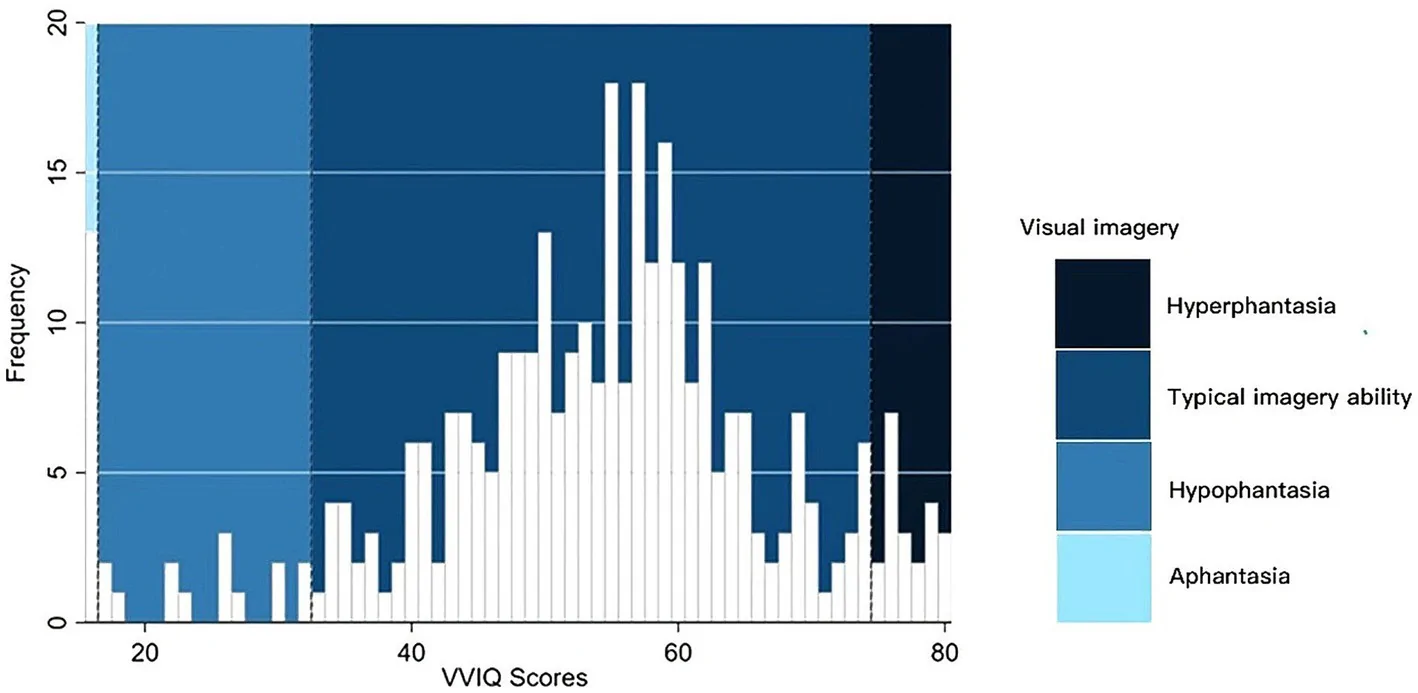
Distribution of Vividness of Visual Imagery Questionnaire (VVIQ) scores across participants. Imagery ability categories (aphantasia, hypophantasia, typical imagery, hyperphantasia) are color coded to show the frequency of participants in each category. Higher scores indicate greater imagery vividness.

The median VVIQ score for the students was 55 (IQR = 46.5–61.5). The distribution of VVIQ scores is shown in Figure 1. Of the 332 participants, 13 were classified as having aphantasia, yielding a prevalence of 3.9% [95% CI (2.3, 6.6)]. Fourteen participants were classified as having hypophantasia [4.2%; 95% CI (2.5, 7.0)]. The majority (*N* = 284) were classified as having typical imagery ability, corresponding to 85.5% [95% CI (81.3, 88.9)]. The remaining 21 participants were classified as having hyperphantasia, representing 6.3% [95% CI (4.2, 9.5)].

Visual recall clarity averaged 3.025 (SD = 0.845; range = 1–5; 95% CI: 2.931–3.118), and item position (1–16) was not associated with recall clarity (*b* = −0.0028, SE = 0.0151, *p* = 0.853, 95% CI: −0.0324 to 0.0268). Answer correctness averaged 11.762 items (SD = 2.256; range = 5–16; 95% CI: 11.494–12.029), and item position showed no effect on correctness (*b* = 0.0713, SE = 0.0520, *p* = 0.171, 95% CI: −0.0309 to 0.1732). Overall exam score averaged 75.184% (SD = 10.146; range = 48–94; 95% CI: 73.984–76.384).

To evaluate the role of visual imagery ability in exam performance, we examined whether VVIQ scores were associated with visual recall clarity during the exam, and how these variables related to exam performance, which were measured by two indicators: the number of correct answers on 16 selected questions and the overall score on all 50 questions, converted to a percentage.

**Figure 2:**
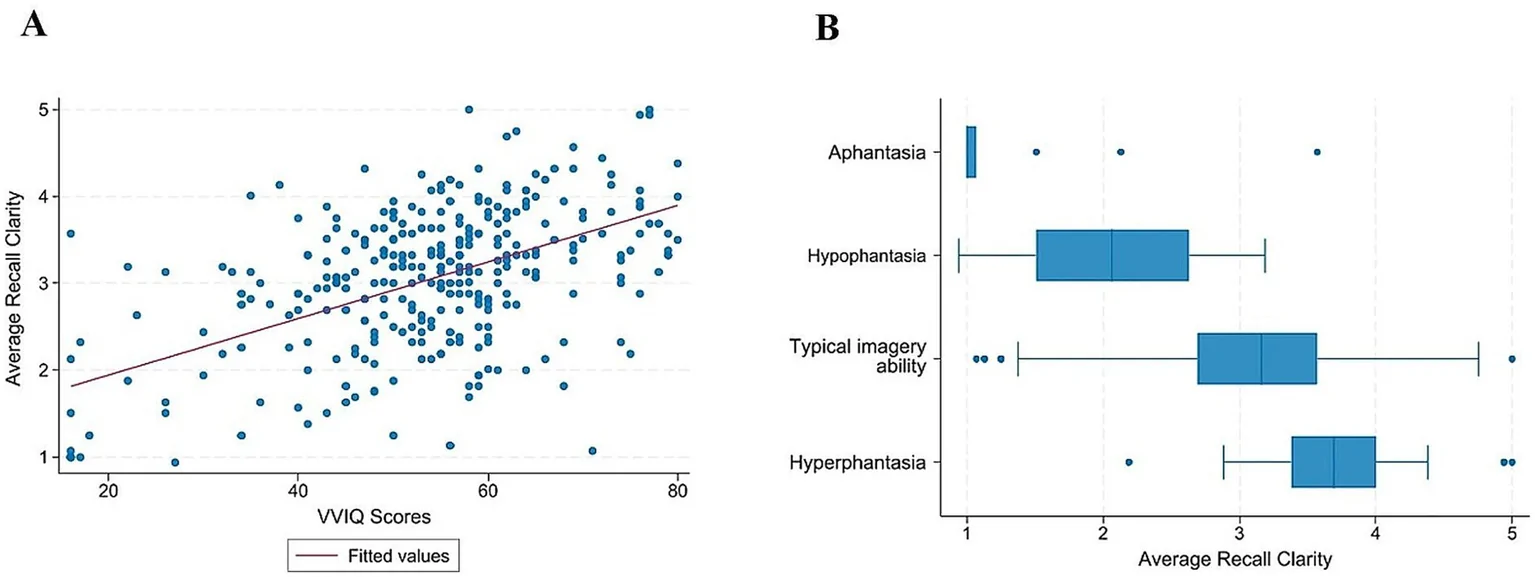
Higher VVIQ scores are associated with greater visual recall clarity. **(A)** Scatterplot with linear fit showing the relationship between average recall clarity and VVIQ scores. **(B)** Boxplots of average recall clarity by imagery ability category.

First, we tested whether VVIQ scores could predict visual recall clarity using simple linear regression. Results showed a significant positive association (*b* = 0.032, *β* = 0.549, *p* < 0.001), indicating that higher VVIQ scores predicted greater clarity of visual recall (Figure 2A). The model explained a substantial proportion of variance (*R*^2^ = 30.19%), which supports that individuals who report more vivid visual imagery (as measured by the VVIQ) also tend to experience clearer visual recall during the exam. The median average recall clarity for the four visual imagery ability categories (aphantasia, hypophantasia, typical imagery ability, and hyperphantasia) were 1.00, 2.07, 3.16, and 3.69, respectively (Figure 2B). These findings indicate that a clearer “mind’s eye” is associated with generating stronger visual mental images during visualization tasks.

**Figure 3:**
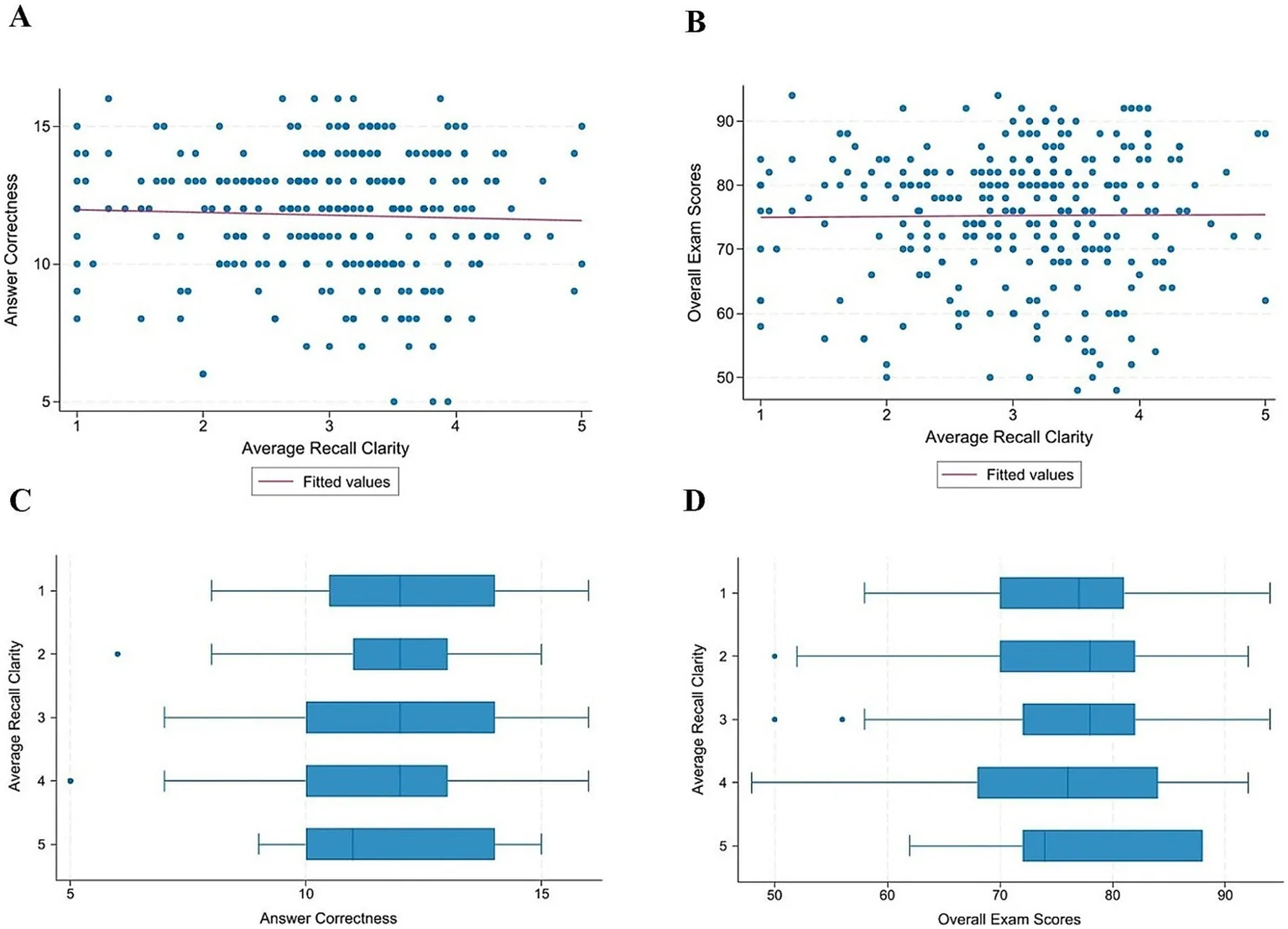
Visual recall clarity does not predict exam performance. **(A,B)** Scatterplots showing the relationship between recall clarity and exam performance. **(C,D)** Exam performance across recall clarity groups.

Next, to evaluate whether students’ subjective visual recall clarity predicts exam performance, we examined the relationship between average recall clarity and two outcomes: answer correctness and overall exam score. Simple linear regressions revealed no significant relationships for either outcome (answer correctness: *b* = −0.049, *β* = −0.037, *p* = 0.538; overall exam score: *b* = 0.102, *β* = 0.009, *p* = 0.886) (Figures 3A,B). The models accounted for only 0.14 and 0.01% of the variance, respectively, suggesting that recall clarity during the exam was not associated with their performance. To further explore this relationship, we grouped average recall clarity into five bins (score = 1–5) and conducted a one-way ANOVA to assess whether students with different levels of recall clarity differed in their exam performance. A one-way ANOVA revealed no statistically significant effect of recall clarity group on exam outcomes (answer correctness: *p* = 0.324; overall exam score: *p* = 0.526). The overall exam scores for the clarity 1–5 groups were 75.8 ± 9.4, 75.1 ± 9.8, 76.1 ± 9.5, 73.6 ± 11.6, and 76.9 ± 9.6, respectively (Figures 3C,D). These results indicate that students’ subjective visual recall clarity is not a reliable predictor of exam outcome.

**Figure 4:**
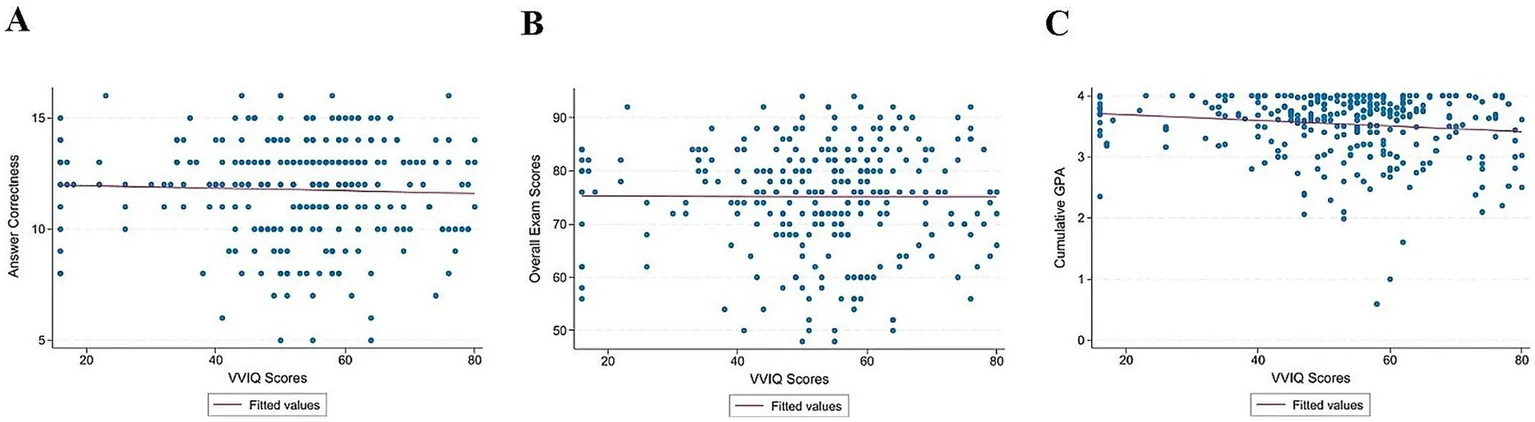
Visual imagery vividness is unrelated to exam performance but weakly associated with cumulative GPA. Scatterplots with linear fits showing the relationship between VVIQ scores and **(A)** answer correctness, **(B)** overall exam score, and **(C)** cumulative GPA.

Then we assessed whether VVIQ scores could directly predict exam performance. Simple linear regressions revealed no significant relationships with exam outcome (answer correctness: *b* = −0.003, *β* = −0.036, *p* = 0.554; overall exam score: *b* = −0.002, *β* = −0.003, *p* = 0.959). The models accounted for 0.13 and 0.00% of the variance, respectively, suggesting that students’ VVIQ scores were not associated with their exam performance (Figures 4A,B). To further explore this relationship, we conducted a one-way ANOVA to assess whether students with different levels of visual imagery ability differed in their exam performance. A one-way ANOVA revealed no statistically significant effect of VVIQ scores on exam outcomes (answer correctness: *p* = 0.894; overall exam score: *p* = 0.803). The overall exam scores for aphantasia, hypophantasia, typical imagery ability, and hyperphantasia were 72.8 ± 10.6, 76.0 ± 7.7, 75.4 ± 10.3, and 74.1 ± 9.6, respectively. These results indicate that visual imagery ability is not a reliable predictor of exam outcome.

To further evaluate the role of visual imagery ability in students’ overall academic success, we tested whether VVIQ scores were associated with cumulative GPA. A simple linear regression revealed a statistically significant association between VVIQ score and cumulative GPA (*b* = −0.005, *β* = −0.127, *p* = 0.026; Figure 4C). We re-estimated this model using heteroskedasticity-robust standard errors, the association remained statistically significant (*b* = −0.005, β = −0.127, *p* = 0.013), indicating that the findings were robust to potential heteroscedasticity. However, the regression coefficient was small, indicating minimal practical significance despite statistical significance. Thus, greater visual imagery ability does not necessarily translate into better long-term academic achievement and may be weakly associated with lower cumulative GPA.

### The recall sources are related to students’ exam performance

**Figure 5:**
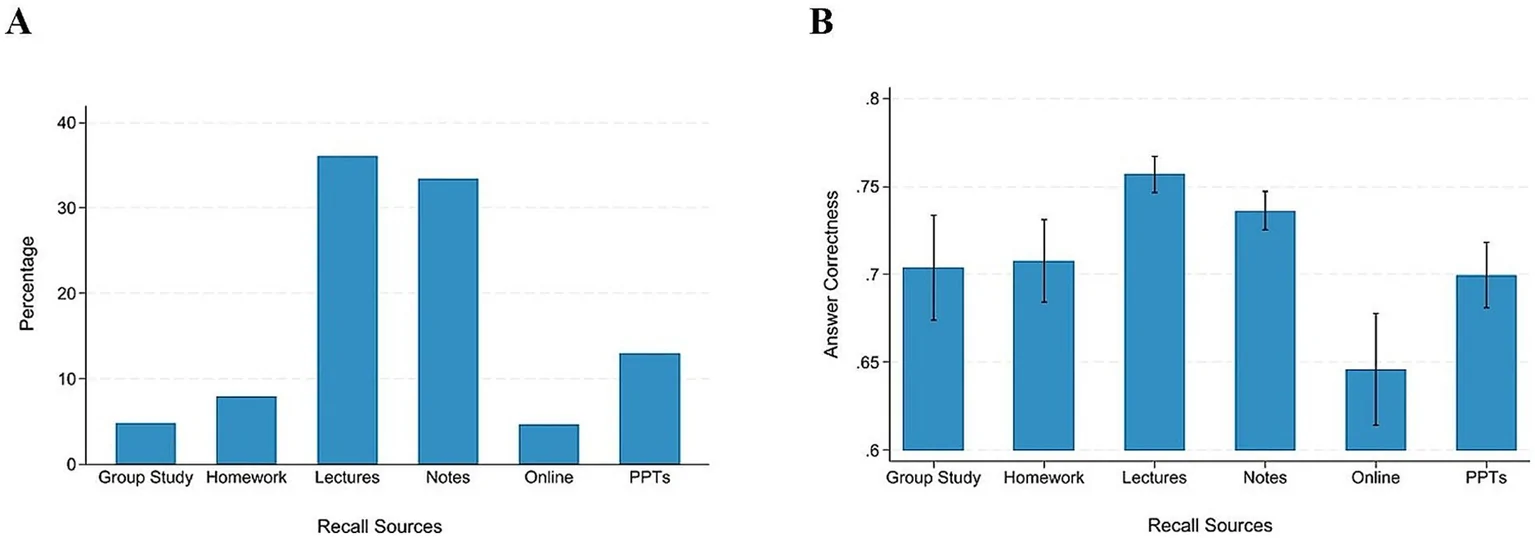
Recall source is associated with differences in answer correctness. Bar graphs showing the percentage **(A)** and answer correctness **(B)** of different study approaches recalled during exams. Error bars represent standard errors of the mean.

We also examined which study approaches students recalled during the exam and which were most effective. As sources of recall, lectures and notes were recalled more frequently (36.07 and 33.44%, respectively), whereas online resources and group study were recalled less often (4.69 and 4.84%, respectively) (Figure 5A). Exam question responses were binary scored (correct = 1, incorrect = 0). Mean scores for each study approach were calculated and compared using one-way ANOVA followed by *post hoc* pairwise comparisons (Figure 5B), which revealed a significant effect of study approach on answer correctness (*F* (5, 4,809) = 3.92, *p* = 0.0015); lectures (*M* = 0.757) had significantly higher scores than online resources (*M* = 0.646). A chi-square test of independence showed a significant association between answer correctness and recall source (*χ*^2^ (5) = 19.56, *p* = 0.002). Adjusted residuals indicated that students who recalled lectures had significantly higher accuracy (residual = 3.08), while those recalling online resources had significantly lower accuracy (residual = −2.95); no other approaches showed significant differences. These results support the interpretation that lecture-based study may be more beneficial than online resources for exam performance.

To examine whether differences among recall-source categories could be explained by question difficulty, we calculated an item difficulty index as the proportion of students answering each item correctly. Item difficulty did not differ significantly across recall-source categories (group study: 0.731 ± 0.172; homework: 0.745 ± 0.198; lectures: 0.745 ± 0.161; notes: 0.736 ± 0.166; online resources: 0.724 ± 0.167; PPTs: 0.731 ± 0.147). A one-way ANOVA revealed no significant effect of recall source on item difficulty, *F* (5, 4,809) = 1.38, *p* = 0.228. Bonferroni-adjusted pairwise comparisons were likewise non-significant. These findings indicate that lecture-recalled items were not systematically easier than items associated with other recall sources, suggesting that item difficulty is unlikely to account for the observed differences among recall-source groups.

### Visual learning preference is weakly linked to recall clarity but does not predict exam performance

**Figure 6:**
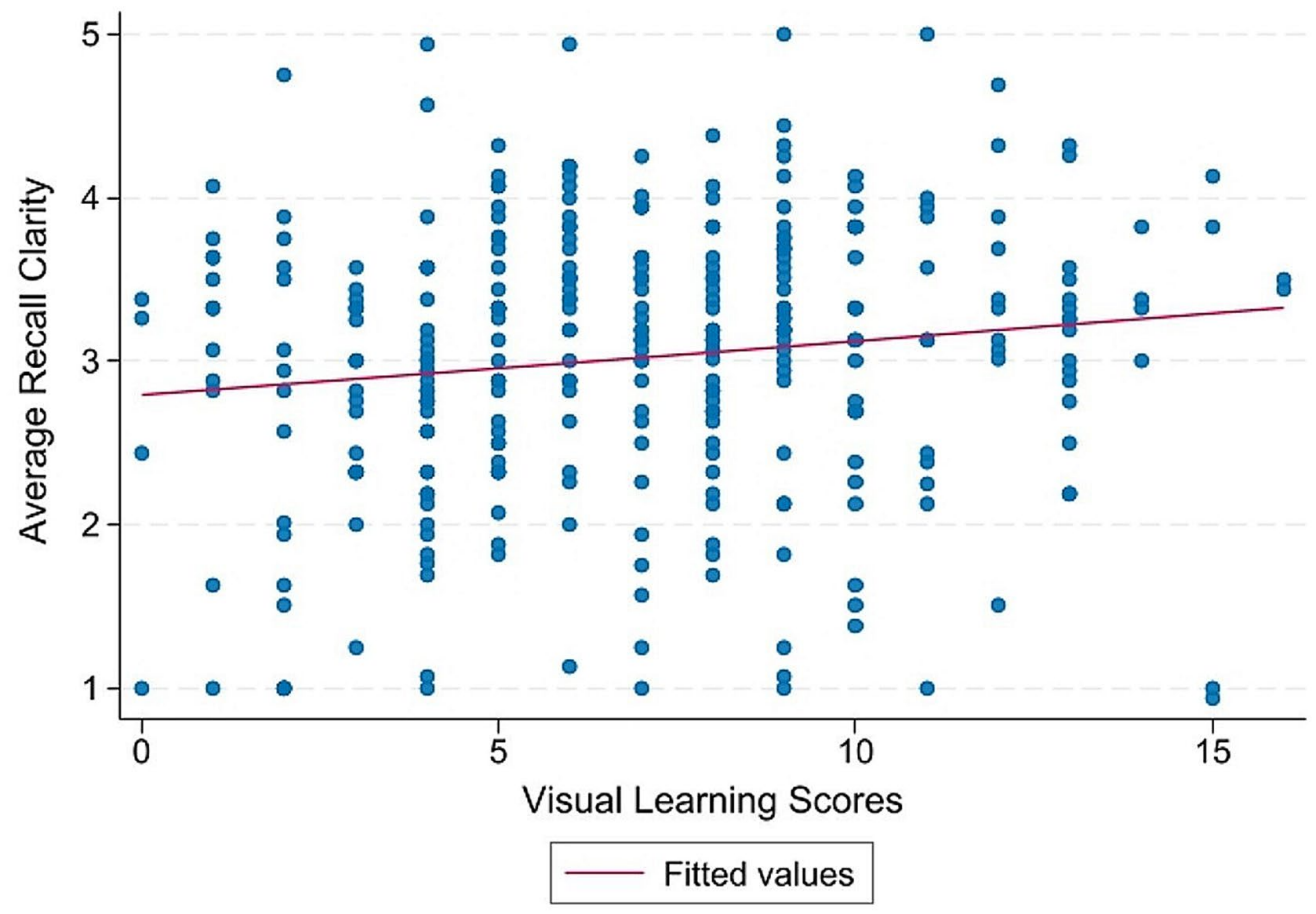
Scatterplot shows a weakly positive relationship between visual learning scores and recall clarity.

The number of visual responses on the completed VARK questionnaire was counted for each participant to represent their degree of visual learning preference (range: 0–16). Higher scores indicate a stronger preference for a visual learning style. A simple linear regression showed a statistically significant association between visual learning score and subjective recall clarity (*b* = 0.033, *β* = 0.136, *p* = 0.016). However, the model explained only 1.85% of the variance in recall clarity, indicating that the effect was of limited practical significance despite its statistical significance (Figure 6). We then assessed whether visual learning scores could predict exam performance; simple linear regression models revealed no significant relationships with exam outcomes (answer correctness: *b* = 0.015, *β* = 0.047, *p* = 0.435; overall exam score: *b* = 0.032, β = 0.011, *p* = 0.853). The models accounted for 0.22 and 0.01% of the variance, respectively, suggesting that students’ preference for visual learning was not associated with their exam performance.

### Study time correlates positively with recall clarity but weakly negatively with exam performance

A regression analysis showed a strong positive relationship between study time after class and recall clarity (*b* = 0.216, *β* = 0.184, *p* = 0.001); the model explained 3.39% of the variance in recall clarity. While statistically significant, study time explained only 3.39% of the variance in recall clarity, indicating a modest effect. We further assessed whether study time predicted exam performance; regression analyses revealed weak negative relationships for exam outcomes (answer correctness: *b* = −0.382, *β* = −0.123, *p* = 0.040; overall exam score: *b* = −1.710, *β* = −0.123, *p* = 0.041). The models accounted for 1.52 and 1.51% of the variance, respectively, indicating that more study time did not necessarily translate into higher exam scores. Some students may have achieved lower scores despite spending more time studying, possibly due to weaker study skills or poorer foundational knowledge.

## Discussion

This study utilized question-level data from a large undergraduate neuroscience course to examine whether individual differences in visual imagery vividness (VVIQ), subjective visual recall clarity during exams, and a preference for visual learning (VARK) translate into superior performance on time-limited, closed-book summative assessments. The main findings are as follows: VVIQ scores and reported recall clarity were moderately and reliably correlated; however, neither visual imagery vividness nor recall clarity predicted answer correctness or overall exam scores. A weak positive association between visual learning preference and recall clarity was observed, with a very small effect size explaining less than 2% of the variance and negligible links to performance. Students’ recall of lectures, a behavioral indicator of alignment with classroom instruction, was associated with higher answer correctness relative to recalling online resources; this association is correlational and should not be interpreted causally. Together, the results refine our understanding of visual imagery in applied educational contexts and support cautious, task-dependent instructional implications.

The lack of correlation between visual imagery vividness and academic performance is in line with recent domain-specific research showing task-dependent benefits of visual imagery (Bainbridge et al., 2020; Keogh et al., 2021). Research in laboratories and specific fields indicates that vivid visual imagery supports encoding and retrieval for the tasks that rely on spatial or pictorial reconstruction (Bobek and Tversky, 2016; Tabi et al., 2021). Our findings contribute to the existing literature (Suggate, 2024) by showing that, in real classroom settings using multiple-choice exams that assess conceptual understanding and application, self-reported visual imagery vividness explains roughly 30% of variance in recall clarity but does not translate into improved exam performance. This pattern suggests that vividness of visual imagery primarily influences the subjective experience of retrieval rather than objective success on heterogeneous, concept-based summative assessments, consistent with findings that imagery benefits are strongest for visuospatial reconstruction tasks and weaker for generalized academic performance (Leopold and Mayer, 2015; Pounder et al., 2022).

Several factors may account for the absence of an association with exam performance. First, subjective visual imagery does not map uniformly onto task demands: many exam items in large undergraduate science courses assess conceptual integration and causal reasoning rather than visuospatial reconstruction (McDaniel et al., 2022). If imagery helps only on a subset of visuospatial items, any benefit will be diluted in heterogeneous exams. Second, subjective measures such as the VVIQ and clarity of visual recall capture introspective reports rather than objective imagery fidelity (Cavazos et al., 2025). Prior work shows that self-reported vividness is inconsistently associated with neural and behavioral measures across contexts (Runge et al., 2017). Individuals with aphantasia or hypophantasia often adopt compensatory strategies (e.g., externalization, verbalization, associative mapping) to achieve performance comparable to peers (Pounder et al., 2022). Thus, the lack of predictive validity for exam scores may reflect both measurement characteristics and strategic flexibility among learners.

We found that recalling from lectures during exams was associated with higher answer correctness and overall exam scores. This suggests that engagement with classroom instruction may be more strongly associated with academic performance than the subjective vividness of mental imagery (Guo et al., 2020). The statistically significant association between VVIQ score and cumulative GPA should be interpreted with caution. Although the regression coefficient reached statistical significance, its magnitude was small, and the model explained only a small proportion of the variance in GPA, indicating minimal practical significance. Therefore, this finding should not be interpreted as evidence that visual imagery ability meaningfully influences long-term academic achievement. Furthermore, in a multivariable regression model including VVIQ score, major, class, and visual learning preference, the association between VVIQ score and cumulative GPA was attenuated and remained of negligible practical significance. This highlights the need to model background variables in multivariable analyses to assess whether visual imagery has an independent association.

The study contributes a novel application of question-level assessment data to examine the relationship between visual imagery, retrieval experiences, and academic performance. First, it links validated measures of individual differences (VVIQ and VARK) to real classroom, question-level assessment data from a large, diverse sample of non-major students (Amaniyan et al., 2020). This approach moves beyond laboratory tasks and small samples that predominate in imagery research and clarifies how subjective imagery maps onto real classroom performance (Zhou et al., 2024). Second, by collecting self-reports of recall source and clarity for randomly sampled items, we could examine not only ability but also the applied strategies during retrieval. Third, the contrast between subjective phenomenology (recall clarity) and objective performance revealed a dissociation that clarifies the basis of visual imagery effects: vivid imagery influences the subjective quality of recall more than it determines objective success on summative exams.

Although the recall clarity measure demonstrated strong internal consistency, it represents a novel self-report measure of state-level imagery during retrieval. Further work is needed to establish its construct validity and relationship to other measures of mental imagery and memory processes. To strengthen causal and practical conclusions, future work should take two directions. First, objective tests of visual imagery, such as behavioral tasks (e.g., imagery interference) or neural measures (e.g., pattern similarity analyses), could be used alongside the VVIQ to assess whether objective imagery fidelity predicts performance in cases where self-reports do not (Runge et al., 2017; Gjorgieva et al., 2023). Second, intervention studies that instruct different strategies (e.g., imagery, note-based rehearsal, or drawing) could test whether teaching imagery or providing external visual supports improves performance on visual compared with nonvisual items (Leopold and Mayer, 2015; Bobek and Tversky, 2016).

Our findings indicate that visual imagery is not a major predictor of exam success. Students with more vivid imagery reported better recall clarity during exams, but this advantage was not reflected in higher answer correctness or overall examination scores. The results suggest that visual imagery may improve the subjective experience of remembering without necessarily enhancing performance on heterogeneous, concept-based summative assessments. Similarly, although a preference for visual learning was associated with recall clarity, the effect was small and is unlikely to be of practical importance for academic achievement.

Students who reported recalling lecture content during the exam answered more questions correctly than those relying on online resources. This correlation does not mean causation, but it suggests that learning strategies closely aligned with classroom instruction may better support exam performance than reliance on external materials alone. At the same time, the present findings provide little evidence that encouraging visual imagery, by itself, is likely to improve exam outcomes. Instead, its effectiveness probably depends on the nature of the learning task. It may provide meaningful benefits for the tasks requiring visuospatial reasoning or mental reconstruction.

Overall, visual imagery ability was positively associated with recall clarity, whereas both variables showed negligible associations with academic performance. Similarly, although visual learning preference was statistically associated with recall clarity during the examination, the effect size was small, indicating limited practical significance. The findings suggest that academic performance is influenced by a combination of cognitive, motivational, and behavioral factors rather than by individual differences in visual imagery ability or visual learning preference alone.

## Data Availability

The raw data supporting the conclusions of this article will be made available by the authors, without undue reservation.

## Supplementary material

SUPPLEMENTARY FIGURE S1 No obvious pattern is observed in any of the linear regression models (visual inspection), although Model 6 showed statistically significant heteroscedasticity as indicated by the Breusch-Pagan test. Model 1: VVIQ scores predicting visual recall clarity; Model 2: Visual recall clarity predicting answer correctness; Model 3: Visual recall clarity predicting overall exam scores; Model 4: VVIQ scores predicting answer correctness; Model 5: VVIQ scores predicting overall exam scores; Model 6: VVIQ scores predicting cumulative GPA; Model 7: VARK visual learning score predicting visual recall clarity; Model 8: VARK visual learning score predicting answer correctness; Model 9: VARK visual learning score predicting overall exam scores.

SUPPLEMENTARY FIGURE S2 Models 1–5 and 7–9 show approximately normal residuals, whereas Model 6 shows a clear deviation from normality. Model 1: VVIQ scores predicting visual recall clarity; Model 2: Visual recall clarity predicting answer correctness; Model 3: Visual recall clarity predicting overall exam scores; Model 4: VVIQ scores predicting answer correctness; Model 5: VVIQ scores predicting overall exam scores; Model 6: VVIQ scores predicting cumulative GPA; Model 7: VARK visual learning score predicting visual recall clarity; Model 8: VARK visual learning score predicting answer correctness; Model 9: VARK visual learning score predicting overall exam scores.
