# Supplementary material for: Vividness of visual imagery is associated with perceived clarity of recall but not with accuracy during exams
Source: Front Psychol. 2026 Aug 28;17:1867712. doi: 10.3389/fpsyg.2026.1867712 (PMC13561970; doi:10.3389/fpsyg.2026.1867712)
Supplement: Supplementary file 1 [file Table_1.DOCX]

**Supplementary Table S1. Descriptive statistics for the primary study variables**

|  | Mean | SD | Min | Max | [95% conf. interval] |
| --- | --- | --- | --- | --- | --- |
| visual recall clarity | 3.025 | 0.845 | 1 | 5 | 2.931, 3.118 |
| answer correctness | 11.762 | 2.256 | 5 | 16 | 11.494, 12.029 |
| overall exam score | 75.184 | 10.146 | 48 | 94 | 73.984, 76.384 |
| visual learning score | 6.988 | 3.468 | 0 | 16 | 6.613, 7.362 |
