# Supplementary material for: Vividness of visual imagery is associated with perceived clarity of recall but not with accuracy during exams
Source: Front Psychol. 2026 Aug 28;17:1867712. doi: 10.3389/fpsyg.2026.1867712 (PMC13561970; doi:10.3389/fpsyg.2026.1867712)
Supplement: Supplementary file 2 [file Table_2.DOCX]

| **Model** | **Predictor** | **Outcome** | **Residual-versus-fitted plot** | **Q-Q plot** | **Shapiro-Wilk (W, p)** | **Breusch-Pagan (χ², p)** | **White test**  **(χ², p)** |
| --- | --- | --- | --- | --- | --- | --- | --- |
| 1 | VVIQ scores | Visual recall clarity | No obvious pattern | Approximately normal | W = 0.9936,  p = 0.2040 | χ² = 0.39  p = 0.5348 | χ² = 2.12  p = 0.3461 |
| 2 | Visual recall clarity | Answer correctness | No obvious pattern | Approximately normal | W = 0.9687,  p = 0.00001 | χ² = 0.94  p = 0.3314 | χ² = 0.97  p = 0.6151 |
| 3 | Visual recall clarity | Overall exam scores | No obvious pattern | Approximately normal | W = 0.9618,  P = 0.0000 | χ²=0.96  p = 0.3280 | χ² = 1.19  p = 0.5522 |
| 4 | VVIQ scores | Answer correctness | No obvious pattern | Approximately normal | W = 0.9683,  p = 0.00001 | χ² = 0.17  p = 0.6827 | χ² = 1.49  p = 0.4740 |
| 5 | VVIQ scores | Overall exam scores | No obvious pattern | Approximately normal | W = 0.9612,  p = 0.0000 | χ² = 0.21  p = 0.6451 | χ² = 1.06  p = 0.5890 |
| 6 | VVIQ scores | Cumulative GPA | No obvious pattern | Not normally distributed | W = 0.83785,  p = 0.0000 | χ² = 9.50  p = 0.0036 | χ² = 2.52  p = 0.2838 |
| 7 | VARK visual learning score | Visual recall clarity | No obvious pattern | Approximately normal | W = 0.9762,  p = 0.00004 | χ² = 0.00  p = 0.9569 | χ² = 3.42  p = 0.1811 |
| 8 | VARK visual learning score | Answer correctness | No obvious pattern | Approximately normal | W = 0.9713,  p = 0.00002 | χ² = 0.02  p = 0.8860 | χ² = 0.05  p = 0.9760 |
| 9 | VARK visual learning score | Overall exam scores | No obvious pattern | Approximately normal | W = 0.9620,  p = 0.0000 | χ² = 0.10  p = 0.7547 | χ² = 0.43  p = 0.8052 |

**Supplementary Table S2. Diagnostic tests for regression assumptions across all regression models**

**Note. Shapiro-Wilk tests were used to assess the normality of residuals. Breusch-Pagan and White tests were used to assess homoscedasticity. Non-significant p values (p > 0.05) for the Breusch-Pagan and White tests indicate no evidence of heteroscedasticity. Although some Shapiro-Wilk tests were statistically significant, visual inspection of the corresponding Q-Q plots indicated only minor deviations from normality. Given the relatively large sample size, these deviations were not considered substantial. Because the Breusch-Pagan test indicated heteroscedasticity for Model 6 (p = 0.0036), this model was re-estimated using heteroskedasticity-robust standard errors. The substantive conclusions remained unchanged.**
